# Supplementary material for: Long term conservation of DNA at ambient temperature. Implications for DNA data storage
Source: PLoS One. 2021 Nov 11;16(11):e0259868. doi: 10.1371/journal.pone.0259868 (PMC8585539; doi:10.1371/journal.pone.0259868)
Supplement: S1 File — (DOCX) [file pone.0259868.s001.docx]

Supporting Information File S1.

# Gel electrophoresis

## DNA sizing on agarose gels

As controls, before qPCR, we checked the DNA size profiles by gel electrophoresis.

We ran non-denaturing agarose gels (AGE) after heat denaturation (95 °C for 5 min) to evidence single strand breaks. The gels were stained for one hour with ethidium bromide (500 ng/mL) and photographed with a digital imaging device under UV light (Quantum ST4 1000, Vilber Lourmat, Collégien, France). The images were analyzed with the Bio1D image analysis software (Vilber Lourmat, Collégien, France).

It is known that heat-denatured mammalian DNA brought back at ambient temperature contains a small but non negligible proportion of double strands because of internal foldbacks[1]. Moreover, the sizes were measured by reference to non-denatured molecular weight markers. So, while this procedure is sufficient for estimating DNA size, it gives only “apparent” sizes.

**S1 Figure Electrophoresis of DNA samples after degradation at 140 °C, 130 °C, 120 °C,110 °C, 100 °C versus time.**

At each time point of storage (between 2 min and 48 hours) at a given temperature, we determined the length of the molecule corresponding to the maximum of the mass distribution of the DNA population (i.e. to the maximum staining intensity of the gel), L*_max_* as previously described [2]. These sizes are given under the gels.

The samples, for a given temperature were run on several gels so the pictures are composites.

M: molecular weight Thermo Scientific GenRuler 1 kb plus DNA Ladder .

# References

1. Soriano P, Macaya G, Bernardi G. The Major Components of the Mouse and Human Genomes. Eur J Biochem. 1981; 115(2): 235-239. doi: 10.1111/j.1432-1033.1981.tb05228.x

2. Colotte M, Couallier V, Tuffet S, Bonnet J. Simultaneous assessment of average fragment size and amount in minute samples of degraded DNA. Anal Biochem. 2009; 388345–347. doi: 10.1016/j.ab.2009.02.003
