# Supplementary material for: Long term conservation of DNA at ambient temperature. Implications for DNA data storage
Source: PLoS One. 2021 Nov 11;16(11):e0259868. doi: 10.1371/journal.pone.0259868 (PMC8585539; doi:10.1371/journal.pone.0259868)
Supplement: S2 File — (PPTX) [file pone.0259868.s002.pptx]

## Slide 1
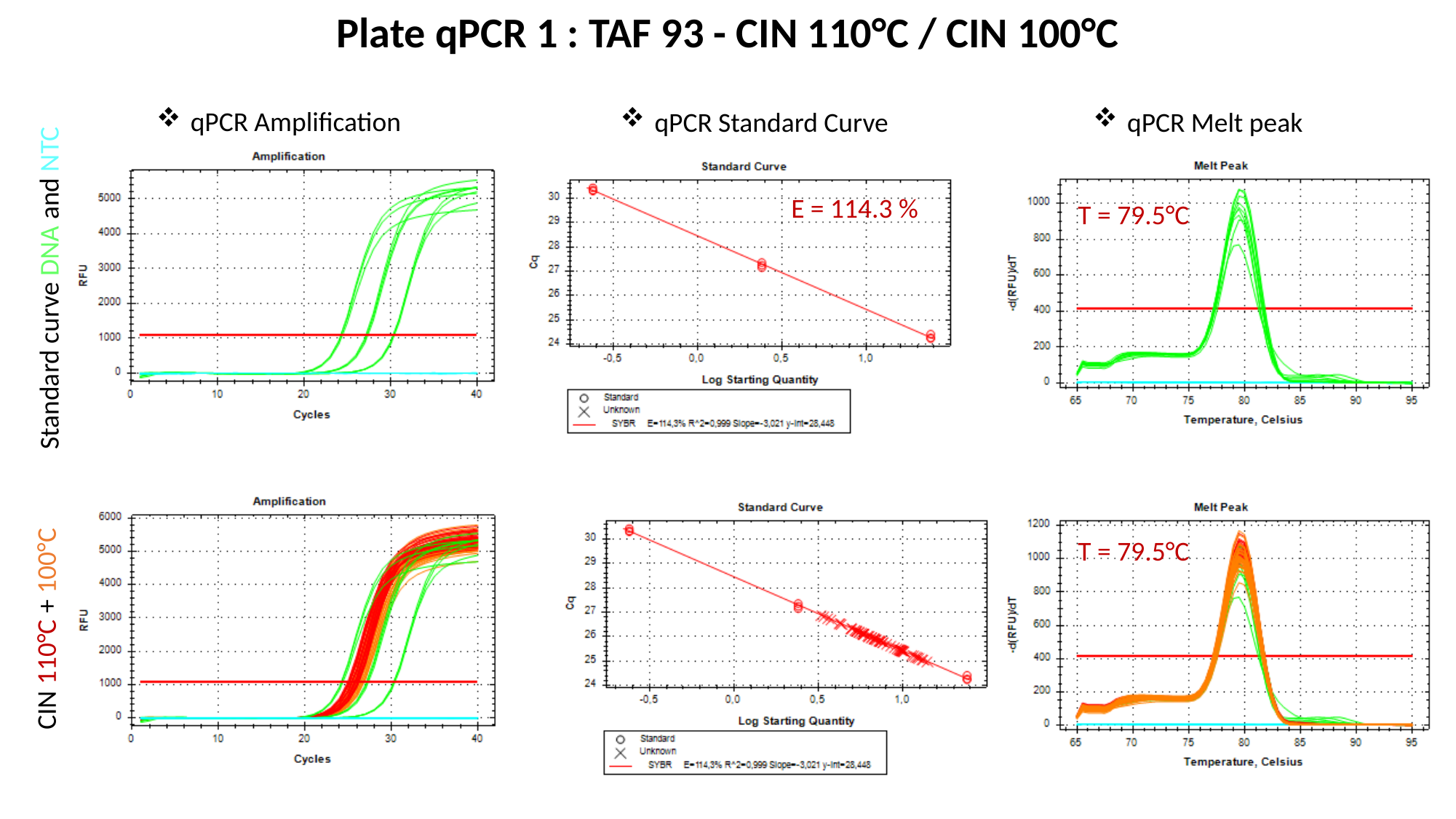

Plate qPCR 1 : TAF 93 - CIN 110°C / CIN 100°C
qPCR Amplification
qPCR Standard Curve
qPCR Melt peak
E = 114.3 %
T = 79.5°C
Standard curve DNA and NTC
T = 79.5°C
CIN 110°C + 100°C

## Slide 2
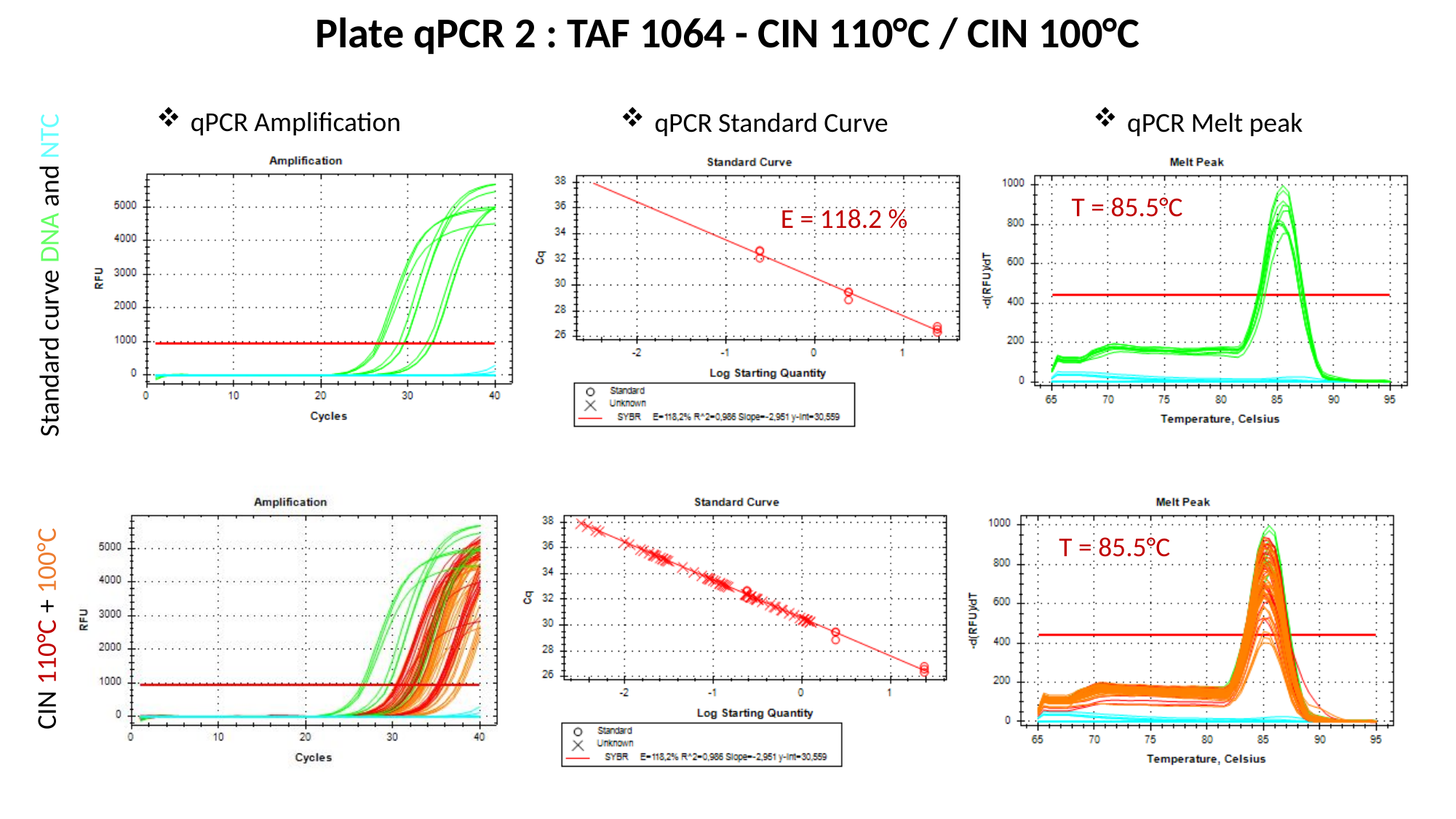

Plate qPCR 2 : TAF 1064 - CIN 110°C / CIN 100°C
qPCR Amplification
qPCR Standard Curve
qPCR Melt peak
T = 85.5°C
E = 118.2 %
Standard curve DNA and NTC
T = 85.5°C
CIN 110°C + 100°C

## Slide 3
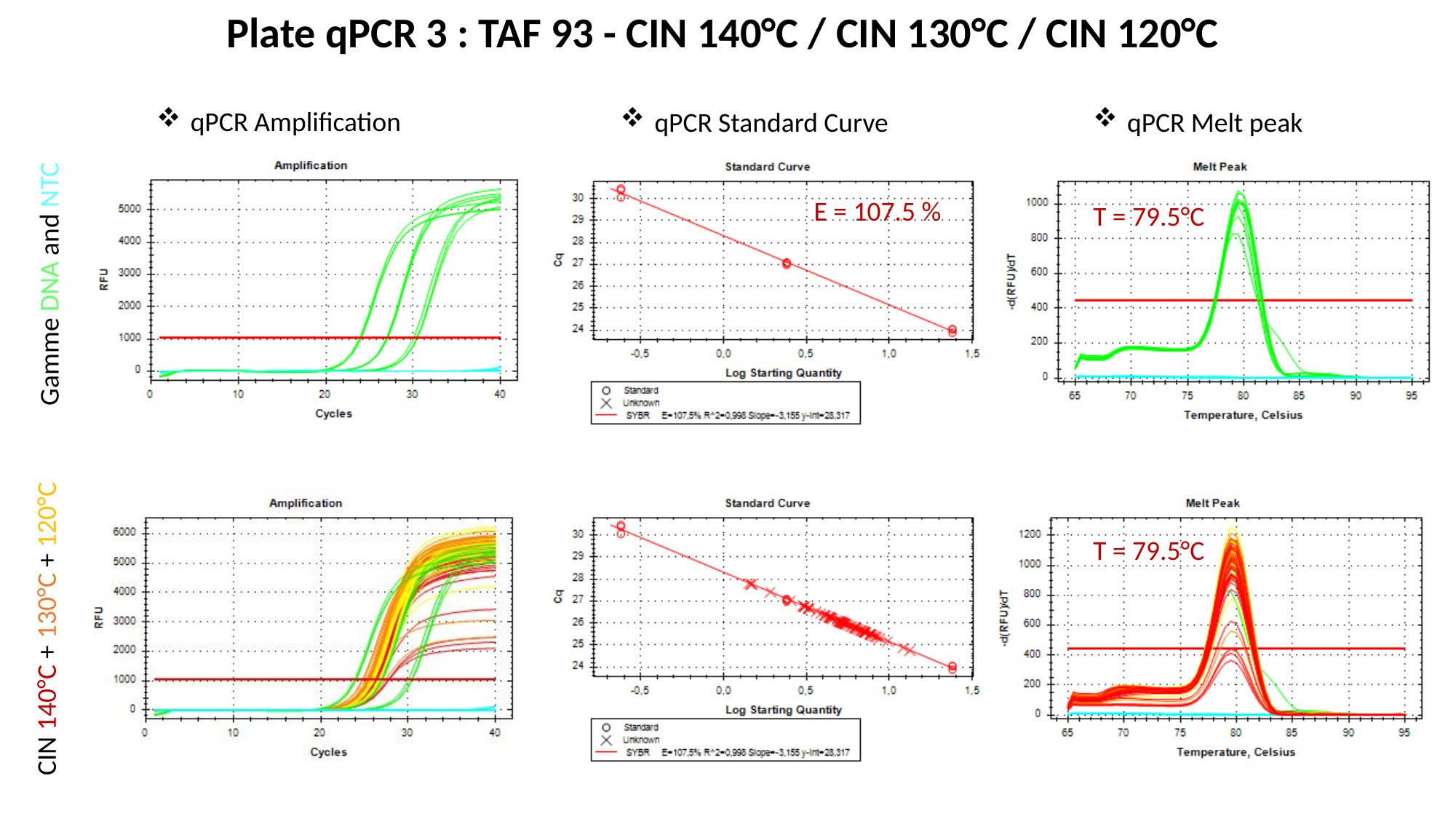

Plate qPCR 3 : TAF 93 - CIN 140°C / CIN 130°C / CIN 120°C
qPCR Amplification
qPCR Standard Curve
qPCR Melt peak
E = 107.5 %
T = 79.5°C
Gamme DNA and NTC
T = 79.5°C
CIN 140°C + 130°C + 120°C

## Slide 4
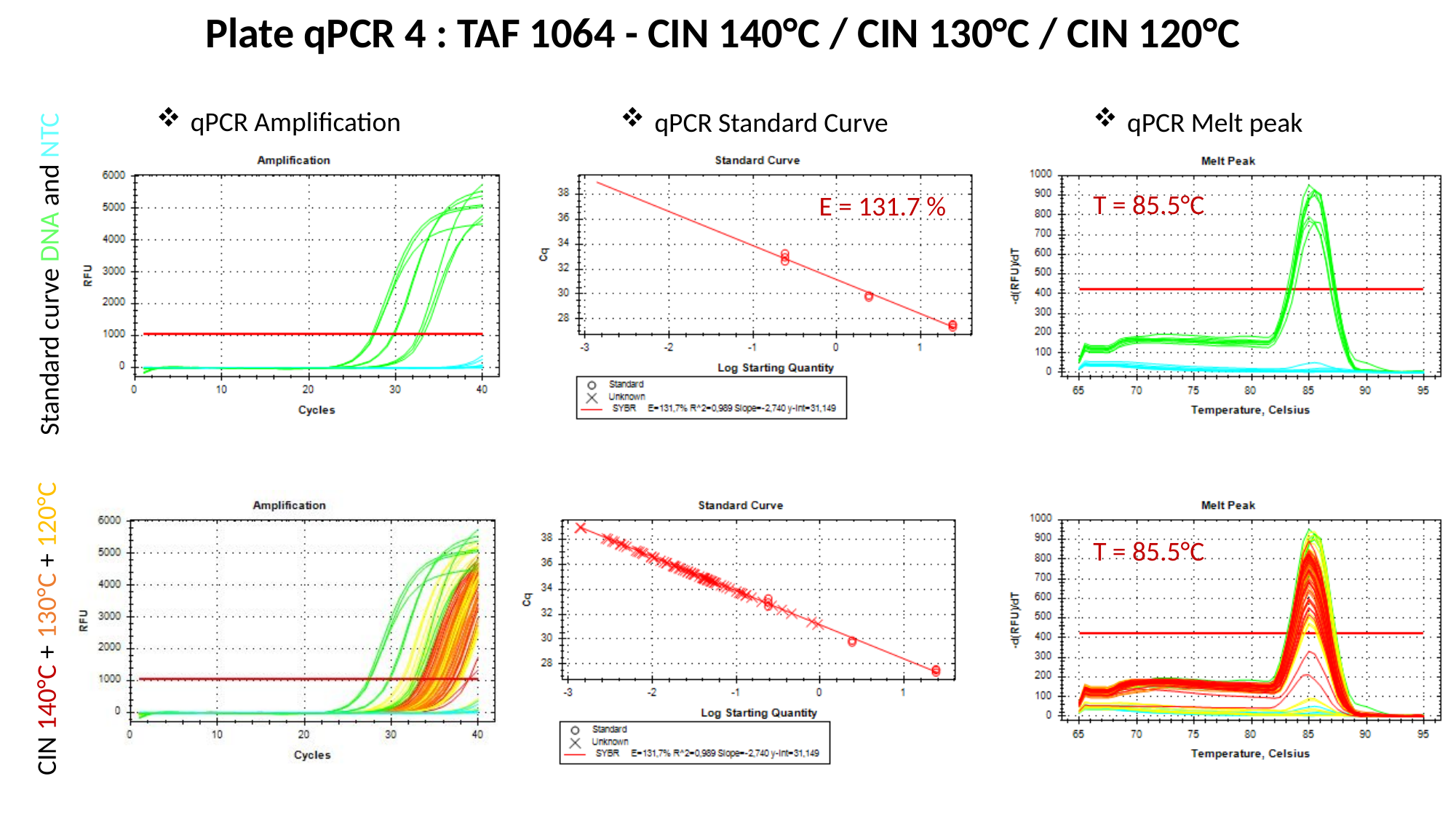

Plate qPCR 4 : TAF 1064 - CIN 140°C / CIN 130°C / CIN 120°C
qPCR Amplification
qPCR Standard Curve
qPCR Melt peak
T = 85.5°C
E = 131.7 %
Standard curve DNA and NTC
T = 85.5°C
CIN 140°C + 130°C + 120°C
